# Direct Quantification of Rare Earth Elements Concentrations in Urine of Workers Manufacturing Cerium, Lanthanum Oxide Ultrafine and Nanoparticles by a Developed and Validated ICP-MS

**DOI:** 10.3390/ijerph13030350

**Published:** 2016-03-22

**Authors:** Yan Li, Hua Yu, Siqian Zheng, Yang Miao, Shi Yin, Peng Li, Ying Bian

**Affiliations:** 1 State Key Laboratory of Quality Research in Chinese Medicine, Institute of Chinese Medical Sciences, University of Macau, Av. Padre Tomás Pereira Taipa, Macau 999078, China; ly77109@163.com (Y.L.); bcalecyu@umac.mo (H.Y.); zsq050@126.com (S.Z.); ymiao777@gmail.com (Y.M.); seamus_alice@163.com (S.Y.); 2 Shanghai Institute of Occupational Safety and Health (SIOSH), 369 North Chengdu Road, Shanghai 200041, China

**Keywords:** rare earth elements (REEs), cerium and lanthanum oxide nanoparticles, inductively coupled plasma-mass spectrometry (ICP-MS), urine, human bio-monitoring

## Abstract

Rare earth elements (REEs) have undergone a steady spread in several industrial, agriculture and medical applications. With the aim of exploring a sensitive and reliable indicator of estimating exposure level to REEs, a simple, accurate and specific ICP-MS method for simultaneous direct quantification of 15 REEs (^89^Y, ^139^La, ^140^Ce, ^141^Pr, ^146^Nd, ^147^Sm, ^153^Eu, ^157^Gd, ^159^Tb, ^163^Dy, ^165^Ho, ^166^Er, ^169^Tm, ^172^Yb and ^175^Lu) in human urine has been developed and validated. The method showed good linearity for all REEs in human urine in the concentrations ranging from 0.001–1.000 μg∙L^−1^ with *r^2^* > 0.997. The limits of detection and quantification for this method were in the range of 0.009–0.010 μg∙L^−1^ and 0.029–0.037 μg∙L^−1^, the recoveries on spiked samples of the 15 REEs ranged from 93.3% to 103.0% and the relative percentage differences were less than 6.2% in duplicate samples, and the intra- and inter-day variations of the analysis were less than 1.28% and less than 0.85% for all REEs, respectively. The developed method was successfully applied to the determination of 15 REEs in 31 urine samples obtained from the control subjects and the workers engaged in work with manufacturing of ultrafine and nanoparticles containing cerium and lanthanum oxide. The results suggested that only the urinary levels of La (1.234 ± 0.626 μg∙L^−1^), Ce (1.492 ± 0.995 μg∙L^−1^), Nd (0.014 ± 0.009 μg∙L^−1^) and Gd (0.023 ± 0.010 μg∙L^−1^) among the exposed workers were significantly higher (*p* < 0.05) than the levels measured in the control subjects. From these, La and Ce were the primary components, and accounted for 88% of the total REEs. Lanthanum comprised 27% of the total REEs while Ce made up the majority of REE content at 61%. The remaining elements only made up 1% each, with the exception of Dy which was not detected. Comparison with the previously published data, the levels of urinary La and Ce in workers and the control subjects show a higher trend than previous reports.

## 1. Introduction

Rare earth elements (REEs) are widely used in areas of agriculture, national defense, new energy, biological medicine, aerospace and the nuclear industry and daily life [1,2], such as fertilizers, automotive catalysts, luminescent materials, high-performance permanent magnets, contrast agents in biomedical imaging, antitumor medicine, nuclear radiation detector [3,4]. Wide utilization implies the current- and growing-spread of REEs in environmental and occupational exposure. The literature from animal studies and limited data from human occupational exposures suggest that REEs have redox reactivity, involving ROS formation, lipid peroxidation and modulation of antioxidant activities, have ephro- and hepato-toxicity, and can induce tissue-specific bioaccumulation [5,6,7,8].

To assess the potential risk to human health, it is necessary to investigate the exposure level of REEs, namely “source emissions environmental concentration-exposure human biological monitoring- health effects surveillance”. In this continuum, biological monitoring is an accurate and reliable complement to environmental monitoring [9,10,11,12]. Biological monitoring of exposure integrates the absorption incurred from all sources and routes of exposure [13,14]. Metal levels in biological specimens (sputum, blood, urine, hair, nails, *etc.*) can reflect the total exposure from all possible sources based on some reports [4,15,16,17]. Compared with other biological specimen, urine is commonly used for the direct analysis due to its less invasive, easily available, simple mode of collection, storage and sample preparation [18]. Urine is not only an excretory medium, but also a biological sample for assessment of renal functions [19,20,21,22]. The urinary REEs can be quantified quickly and have been widely used to directly reflect the recent human environmental exposure [5].

In order to monitor the levels of the REE(s) in various tissue fluids, the effective methods for sample preparation and determination are necessary [23,24,25]. Currently, the techniques for simultaneous determination of multiple trace elements in human body mainly include inductively coupled plasma atomic emission spectroscopy (ICP-AES), neutron activation analysis, isotope dilution thermal ionization mass spectrometry (ID-TIMS). These techniques have made a marked improvement in the sensitivity, but their detection limits are still unsatisfactory. In 1983, inductively coupled plasma mass spectrometry (ICP-MS) was introduced as a commercially available system with great progress and currently used for a wide range of applications [26]. Today, ICP-MS has become one of the most effective techniques for simultaneous determination of multiple trace or ultra-trace elements (e.g., REEs) in human biological samples, high-purity materials, and geological samples [1,27,28,29]. Among several analytical techniques used to determine the concentration of REEs in urine, ICP-MS technology has the rapid, quasi-simultaneous, multi-element detection capabilities, low detection limits and high sensitivity. It has been used in the quantitative analysis of the individual elements, qualitative and semi-quantitative analysis of all the elements present, and analysis of isotopic ratios [20,21,30,31,32,33,34,35,36,37]. However, the oxide/hydroxide ions formed by light REEs can affect the ICP-MS determination of heavy REEs. Thus, more attention should be focused on the spectral interferences and matrix effects. Spectral interferences occur when two or more molecular or atomic species have the same nominal mass-to-charge ratio so the signal at that mass cannot be resolved [38]. Unlike spectral interferences, matrix effects can not only overlap or enhance the signal, but also cause many physical/chemical effects [39,40]. Therefore, in some complex samples, a number of unexpected interferences may arise, confusing spectra and increasing the risk of erroneous quantification [41,42].

Human urine contains a high proportion of total dissolved solids (TDS) and salt, the TDS may lead to signal suppression and salts often build up on the cones and torch of the ICP-MS instrument after introduction of even a few milliliters of sample [23,42]. Therefore, matrix simplification of urine samples by dilution and/or digestion is often required before analysis to reduce the effects of polyatomic interferences, matrix-induced signal suppression and carbon-enhanced ionization effects in the plasma. Traditional methods (e.g., sample digestion) require extensive sample preparation, which may increase the chance of contamination or loss of sample, thus increasing experimental uncertainty.

The primary aim of this work was to explore a sensitive and reliable indicator of exposure level to rare earth elements. This tool could be used to enhance the health risk assessment and management of workers manufacturing cerium, lanthanum oxide ultrafine and nanoparticles. In this study, an ICP-MS method for quantification of 15 REEs (Y, La, Ce, Pr, Nd, Sm, Eu, Gd, Tb, Dy, Ho, Er, Tm, Yb and Lu) concentrations in diluted human urine was developed and validated. This method was then applied for the determination of urinary samples obtained from 8 control subjects and 23 workers that manufacture cerium and lanthanum oxide ultrafine and nanoparticles.

## 2. Materials and Methods

### 2.1. Sampling

The urine samples were collected in metal-free polypropylene containers and stored at −20 °C. Prior to sample collection, the time of sampling and working hours were collected for the biological monitoring protocol. The exposed subjects investigated (*n* = 23) were the workers employed in a professional enterprise engaged in the manufacture and sale of rare earth powder products. The primary products are cerium, lanthanum oxide ultrafine and nanoparticles—the particle diameters ranged from 0.05 to 0.8 μm. The control subjects investigated (*n* = 8) were the support staff and management personnel from the same enterprise. All of the subjects (*n* = 31) were informed that their urine would be used for REEs determination and agreed to participate in this study. This project financial supported by University of Macau Research Grant, and the project has been approved by Ethical Committee of the University Board, code number “MYRG106 (Y1-L3)-ICMS13-BY”.

### 2.2. Reagents

Nitric acid (Trace SELECT^®^ Ultra) was purchased from Sigma Chemicals Ltd. (St. Louis, MO, USA). The rare earth elements standard solution containing Y, La, Ce, Pr, Nd, Sm, Eu, Gd, Tb, Dy, Ho, Er, Tm, Yb and Lu (100 mg∙L^−1^ for each) was purchased from Shanghai Institute of Quality Inspection and Technical Research (Shanghai, China). Standard Tune B iCAP Q solution contaning Ba, Bi, Ce, Co, In, Li, and U (1.0 μg∙mL^−1^ each) was purchased from Thermo Fisher Scientific (Bremen, Germany). Water with a resistivity of 18.2 M∙cm^−1^ was prepared using a Milli-Q system (Millipore, S.A., St. Quentin Yvelynes, France) and used throughout this work.

### 2.3. Instrumentation

Rare earth element determination was performed by an iCAP™ Q ICP-MS (Thermo Fisher Scientific, Bremen, Germany), typical operating parameters are given in Table 1. Urine samples were introduced by an auto sampler CETAC ASX-520 (CETAC Technologies, Inc., Omaha, NE, USA). Tuning was performed daily using the standard auto tune parameters. Data acquirement and analysis were performed with the software of Qtegra™ Intelligent Scientific Data Solution™ (Qtegra, version 2.4.1800.192).

### 2.4. Sample Preparation and Quality Control

Matrix-matched calibration curves are widely used for the analysis of biological samples to account for matrix effects in inorganic mass spectrometry. In this study, a diluted base urine sample (20-fold dilution with 2% HNO_3_) was used for matrix matching the calibration standards, which ranged from 0.001 to 1.000 μg∙L^−1^. The elements with no isobaric interferences were determined. The monitored elements were ^89^Y, ^139^La, ^140^Ce, ^141^Pr, ^146^Nd, ^147^Sm, ^153^Eu, ^157^Gd, ^159^Tb, ^163^Dy, ^165^Ho, ^166^Er, ^169^Tm, ^172^Yb and ^175^Lu in standard mode. Calibration curve for each element was constructed by plotting the concentration of individual element as a function of signal intensity.

For quality assurance and control, blank spikes (0.01, 0.1 and 1.0 μg∙L^−1^ for all REEs) and standard solution (QC, 0.1 μg∙L^−1^ for all REEs) were used during analyses. The intra-day (CV% (1)) and inter-day (CV% (2)) precisions were defined as the relative standard deviation (RSD) of six replicates of QC sample within 1 day or the QC sample on five separate days, respectively. The recovery was estimated by comparing the determined concentrations of individual element with that of spiked concentrations in samples. The limits of detection (LOD) and limit of quantification (LOQ) for REEs were determined as three times and 10 times of the standard deviation from 11 independent analyses of the base urine. The human urine samples were diluted 20-fold with 2% HNO_3_ before ICP-MS analysis.

### 2.5. Statistical Analysis

All experiments were performed in triplicate. The data were analyzed with IBM SPSS Statistics 22.0 software package. The concentration of 15 REEs are presented as mean ± S.D, median, range and 25th–75th percentile. Values under the LOD were substituted with half of LOD in the computation of means [43]. Variance between the control subjects and the exposed workers was evaluated by Student’s *t*-test and one-way analysis of variance (one-way ANOVA), respectively. A value of *p* < 0.05 was considered significant for all tests.

## 3. Results and Discussion

### 3.1. Method Validation

Current conventions in ICP-MS for quantitative analyses of clinical samples include internal standardization, use of collision/reaction cells and matrix-matched calibrations. Normally, internal standards are used to correct for short term and long-term drift, while reaction/collision cells and matrix-matched calibration curves can help account for spectral and non-spectral interferences [44,45,46]. However, it should not be overlooked that the strategies themselves can impact the accuracy of the results.

In this study, internal standards were examined and the results show no significant difference compared to the results without the internal standard (data not shown). Additionally, uncertainty of ^157^Gd determination usually depends on the concentration of ^141^Pr in the analyte, when the concentration of Pr/Gd ratio increases, it increases the measurement error. Some limited reports show that when the value of ^141^Pr^16^O/^157^Gd > 100, it need a mathematic calibration [47,48,49]. Based on these, we developed and validated an ICP-MS method to investigate the concentration and distribution of 15 REEs in human urine samples. The intra-day and inter-day precisions ranged from 0.43% to 1.28% and from 0.41% to 0.85%, respectively (as summarized in Table 2). Recoveries ranged from 93% to 103% with relative percent differences less than 6.2% for duplicate samples. The LODs and LOQs for the REEs ranged from (0.009–0.011 μg∙L^−1^), (0.029–0.037 μg∙L^−1^), respectively. The results obtained suggest that the method developed in this work is simple, accurate and selective with good reproducibility.

### 3.2. Comparison of the Urinary REEs Concentrations between the Exposed Workers and the Control

The descriptive statistics for REEs in the 31 urine samples of all investigated subjects are presented in Table 3. The 15 REEs were quantitatively determined and reported as mean ± S.D. and confidence interval (95% CI). In the exposed workers’ urine samples, the urinary concentrations of La (1.234 ± 0.626 μg∙L^−1^) and Ce (1.492 ± 0.995 μg∙L^−1^) were significantly higher than other elements, followed by Y (0.031 ± 0.042 μg∙L^−1^), Gd (0.023 ± 0.010 μg∙L^−1^) and Pr (0.020 ± 0.013 μg∙L^−1^). The levels found in the workers ranged from (0.039–2.517 μg∙L^−1^) and (0.331–3.838 μg∙L^−1^) for La and Ce, respectively. The results of the Students *T* test and the one-way ANOVA confirm that the concentrations of (La, Ce, Nd, Gd) in the urine of exposed workers were significantly elevated compared to the controls.

**Figure 1:**
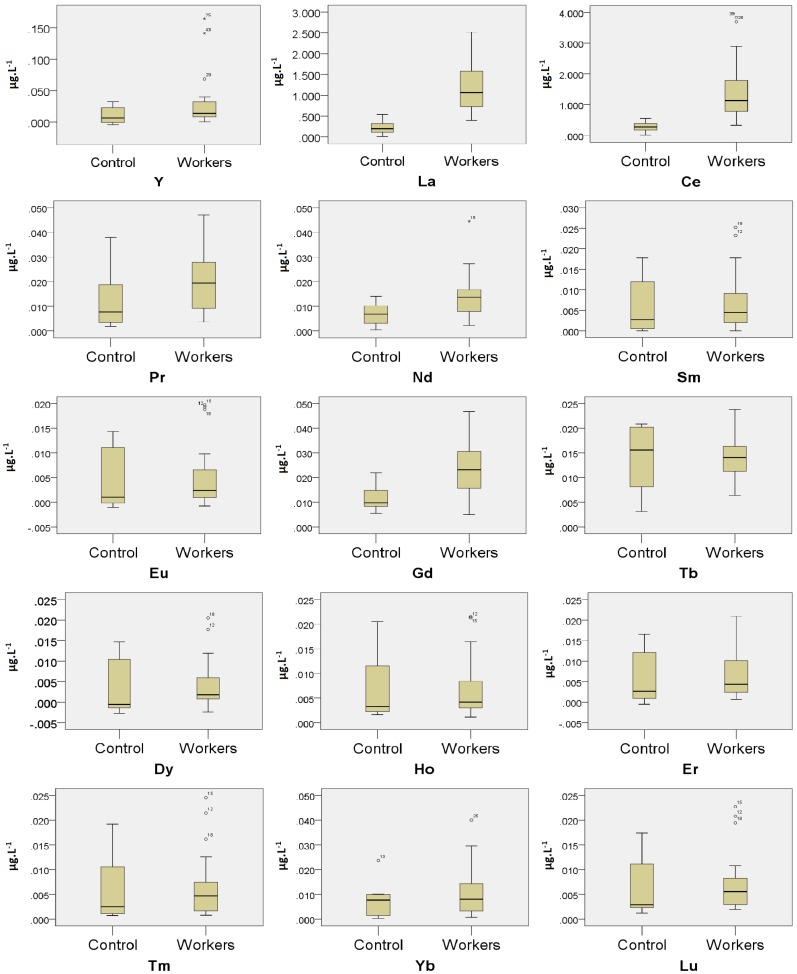
Box and whisker plots for the sum of Y, La, Ce, Pr, Nd, Sm, Eu, Gd, Tb, Dy, Ho, Er, Tm, Yb and Lu concentration levels respectively depending on the control subjects (*n* = 8) and the exposed workers (*n* = 23) who mainly manufacturing cerium, lanthanum oxide ultrafine and nanoparticles.

Box and whisker plots (Figure 1) show the sum of REE concentrations for the control subjects (*n* = 8) and the exposed workers (*n* = 23). There were also some outliers, shown in the box and whisker plots of Y, Ce, Nd, Sm, Eu, Dy, Ho, Tm, Yb, Lu. Preliminary occupational epidemiology analysis shows that these outliers maybe related to the factors such as age, work time and operating post in the workplace. This emphasizes the importance of the multiple factors that contribute to REE exposure. Future research should investigate the role of these factors.

### 3.3. The Distribution Pattern of 15 REEs Concentrations in Urinary Samples

**Figure 2:**
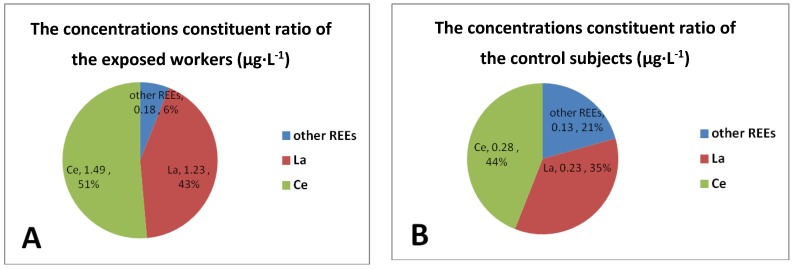
Constituent ratio of the exposed workers (**A**) and the control subjects (**B**).

In order to explore the distribution of the 15 REEs in urine, we performed a constituent ratio analysis of all REEs urinary levels and potential explanatory variables among the occupationally exposed workers and the control subjects based on the concentrations mean value, respectively. Figure 2A shows that the concentrations of La and Ce in the exposed workers were the primary component, together accounting for 94 percent of the total REEs. Lanthanum comprised 43% of the total REEs while Ce made up the majority of REE content at 51%. The remaining elements only made up 6% total. Figure 2B illustrates that the concentrations of REEs in the control subjects, La was 35%, Ce was 44%, other elements equal to 21% total. 

### 3.4. Comparison of the Urinary REEs Concentrations with Other Published Data

Table 4 shows a comparison of our results with several earlier reports on the REEs in general population urine. Liu, *et al.*’s study on the determination of REEs in 19 human urine samples by ICP-MS used HNO_3_ + HClO_4_ wet digestion for sample [50]. In Hao, *et al.*’s cross-sectional study of the urinary REEs concentrations was undertaken in the Baiyun Obo deposit mining area, which is the world’s largest rare earth elements deposit, the investigated subjects were not occupationally exposed population but the general adult population living in the area [3]. In this study, the method does not require labor-intensive digestions and the investigated subjects all employed in a professional enterprise engaged in the manufacture and sale of rare earth powder products.

Comparison with the previously published data, there is a trend that the levels of urinary La and Ce reported as median in workers (1.066 μg∙L^−1^, 1.134 μg∙L^−1^) and control subjects (0.194 μg∙L^−1^, 0.280 μg∙L^−1^) are higher than the general population reported by Liu, *et al.* (0.036 μg∙L^−1^, 0.064 μg∙L^−1^) and Hao *et al.* (0.079 μg∙L^−1^, 0.089 μg∙L^−1^), respectively.

## 4. Conclusions

In summary, an ICP-MS method for simultaneous direct quantification of 15 REEs concentrations in human urine was developed and validated, this assay is simple, accurate, specific and with good reproducibility. By using this method, the concentrations of 15 REEs in 31 urine samples obtained from the control subjects and occupationally exposed workers. The results suggested that the urinary levels of La, and Ce among the workers were significantly enriched compared to those levels measured in the control subjects, the general population and the subjects from REEs deposit mining area. Further research conducted on REEs in occupationally exposed workers should focus on the multiple factors that contribute to REE exposure. More studies of urine, other matrices, or other methods, *etc.* should be done.

## Figures and Tables

**Table 1:** Inductively Coupled Plasma-Mass Spectrometry (ICP-MS) operation parameters.

Parameter	Values
RF Power	1550 W
Focus Lens	21.00
Wash Time	45 s
Sample Uptake Time	45 s
Read Delay	0
Plasma Gas	Ar
Aux. Ar Flow	0.8 L∙min^−1^
Nebulizer Ar Flow	0.9 L∙min^−1^
Cool Ar Flow	14 L∙min^−1^
Additional Gas Flow	0
Dwell Time per Isotope	10 ms
Sweeps/Reading/Number of Sweep	10
Pump Rate	40 rpm
Extraction Lens 1 Negative	−196.5 V
Extraction Lens 1 Positive	−0.05 V
Extraction Lens 2	−187.7 V
Sample Depth	5 mm
Interface Cones	Pt
Measure Mode	STD
^140^Ce^16^O/^140^Ce	1.69% (Tuned Intensity)

**Table 2:** Urine multi-elementary analytical validation (μg∙L^−1^).

Element	*r^2^*	LOD	LOQ	CV% (1)	CV% (2)
^89^Y	1.000	0.010	0.034	0.43	0.59
^139^La	0.997	0.009	0.030	0.51	0.56
^140^Ce	0.999	0.009	0.029	1.18	0.79
^141^Pr	1.000	0.010	0.034	0.75	0.61
^146^Nd	1.000	0.011	0.037	0.58	0.65
^147^Sm	1.000	0.010	0.031	0.51	0.62
^153^Eu	1.000	0.010	0.034	0.59	0.54
^157^Gd	0.999	0.010	0.034	0.54	0.51
^159^Tb	1.000	0.010	0.034	0.93	0.59
^163^Dy	0.999	0.010	0.035	0.64	0.41
^165^Ho	0.999	0.010	0.033	0.82	0.57
^166^Er	0.999	0.010	0.033	0.75	0.54
^169^Tm	0.999	0.010	0.031	0.75	0.59
^172^Yb	0.999	0.010	0.033	1.19	0.79
^175^Lu	0.998	0.010	0.033	1.28	0.85

Notes: *r*^2^: correlation coefficient; LOD: limit of detection; LOQ: limit of quantification; CV% (1) = intra-day precision; CV% (2) = inter-day precision.

**Table 3:** Urinary rare earth elements (REE) levels in the exposed workers and control subjects (μg∙L^−1^, *n* = 31).

	Control (*n* = 8)			Workers (*n* = 23)		
Element	% (>LOD)	Mean ± S.D. (μg∙L^−1^)	95% CI (μg∙L^−1^)	% (>LOD)	Mean ± S.D. (μg∙L^−1^)	95% CI (μg∙L^−1^)
^89^Y	62.5	0.013 ± 0.011	0.004–0.023	87.0	0.031 ± 0.042	0.013–0.049
^139^La *	100.0	0.225 ± 0.170	0.083–0.367	100.0	1.234 ± 0.626	0.963–1.505
^140^Ce *	100.0	0.281 ± 0.171	0.137–0.424	100.0	1.492 ± 0.995	1.061–1.922
^141^Pr	62.5	0.014 ± 0.011	0.005–0.023	95.7	0.020 ± 0.013	0.015–0.026
^146^Nd *	62.5	0.009 ± 0.003	0.006–0.011	87.0	0.015 ± 0.009	0.011–0.019
^147^Sm	62.5	0.008 ± 0.004	0.004–0.012	65.2	0.009 ± 0.006	0.006–0.011
^153^Eu	50.0	0.008 ± 0.003	0.005–0.011	56.5	0.008 ± 0.004	0.006–0.010
^157^Gd *	100.0	0.012 ± 0.006	0.007–0.016	100.0	0.023 ± 0.010	0.019–0.028
^159^Tb	87.5	0.015 ± 0.006	0.009–0.020	100.0	0.014 ± 0.005	0.012–0.016
^163^Dy	50.0	0.008 ± 0.003	0.005–0.011	56.5	0.008 ± 0.004	0.006–0.010
^165^Ho	62.5	0.009 ± 0.006	0.004–0.014	73.9	0.008 ± 0.005	0.006–0.010
^166^Er	50.0	0.009 ± 0.004	0.005–0.013	65.2	0.009 ± 0.005	0.006–0.011
^169^Tm	50.0	0.009 ± 0.004	0.005–0.013	56.5	0.009 ± 0.005	0.006–0.011
^172^Yb	62.5	0.010 ± 0.006	0.005–0.015	65.2	0.013 ± 0.010	0.009–0.016
^175^Lu	50.0	0.009 ± 0.004	0.005–0.012	65.2	0.009 ± 0.005	0.006–0.011

Note: * *p* < 0.05 *vs.* control.

**Table 4:** Comparison of the urinary REEs concentrations with other published data.

Element	This Work (*n* = 31, Median, μg∙L^−1^)		LIU Hu-sheng [50] (*n* = 19, Median, μg∙L^−1^)	Zhe Hao [3] (*n* = 128, Median, μg∙L^−1^)
	Workers (*n* = 23)	Control (*n* = 8)		
^89^Y	0.014	0.008	0.030	0.094
^139^La	1.066	0.194	0.036	0.079
^140^Ce	1.134	0.280	0.064	0.089
^141^Pr	0.019	0.008	0.008	0.030
^146^Nd	0.014	0.007	0.045	0.153
^147^Sm	0.006	0.006	0.054	0.040
^153^Eu	0.006	0.006	0.001	0.019
^157^Gd	0.023	0.010	0.007	0.024
^159^Tb	0.014	0.016	0.002	0.015
^163^Dy	0.006	0.006	0.004	0.020
^165^Ho	0.007	0.006	0.002	0.171
^166^Er	0.006	0.006	0.003	0.011
^169^Tm	0.006	0.006	0.001	0.002
^172^Yb	0.008	0.008	0.003	0.007
^175^Lu	0.006	0.006	0.001	0.002
